# Supplementary material for: Strong bottom currents in large, deep Lake Geneva generated by higher vertical-mode Poincaré waves
Source: Commun Earth Environ. 2024 Sep 3;5(1):480. doi: 10.1038/s43247-024-01653-8 (PMC11371635; doi:10.1038/s43247-024-01653-8)
Supplement: Supplementary file 2 — Supplementary Information [file 43247_2024_1653_MOESM2_ESM.pdf]

Supplementary Information

for

Strong bottom currents in large, deep Lake Geneva generated by higher vertical-mode Poincaré waves

Rafael Sebastian Reiss[1,2], Ulrich Lemmin[1], Claire Monin[1,3], David Andrew Barry[1]

[1]Ecological Engineering Laboratory (ECOL), Institute of Environmental Engineering (IIE), Faculty of Architecture, Civil and Environmental Engineering (ENAC), Ecole Polytechnique Fédérale de Lausanne (EPFL), 1015 Lausanne, Switzerland

[2]Present address: Department of Earth Sciences, University of Cambridge, Cambridge CB2 3EQ, UK

[3]Present address: Research Laboratory in Hydrodynamics, Energetics and Atmospheric Environment (LHEEA), Ecole Centrale de Nantes, UMR CNRS 6598, 44321 Nantes, France

Corresponding authors: Rafael S. Reiss (rr704@cam.ac.uk), D. Andrew Barry (andrew.barry@epfl.ch)

Contents of this file

Supplementary Text 1

Supplementary Table 1

Supplementary Figures 1 to 10

Supplementary Movie 1 (file uploaded separately; movie summary given below)

Supplementary Text 1. Estimation of the V2 and V3 Poincaré wave periods in Lake Geneva

In the main text, vertical mode-two (V2) and vertical mode-three (V3) Poincaré waves in stratified Lake Geneva are investigated with field observations and 3D numerical modeling; wave periods around ~14-15.5 h were determined. Below, the V2 and V3 Poincaré wave periods are estimated from classical concepts provided in the literature for a typical summer stratification using a linear, analytical model for a flat-bottomed, elliptical basin, with the non-rotational phase speed obtained by solving the Taylor-Goldstein equation. Following Antenucci and Imberger[1], the dispersion relation for rotationally-modified internal seiches (Kelvin waves and Poincaré waves) in such basins can be approximated as:

$$\frac{\omega}{f} = a_0 + a_1 S + a_2 S^2 + a_3 S^3, \tag{1}$$

where ω is the angular wave frequency and f is the latitude-dependent Coriolis parameter, which is $O(10^{-4}\ s^{-1})$ at the latitude of Lake Geneva. $S = c/(Lf)$ is the Burger number, with the non-rotating phase speed c and a characteristic length scale L, taken as half the length of the basin's major axis. The constants a_0 to a_3 depend on the wave's sense of rotation, the Burger number, the horizontal wave mode, and the horizontal aspect ratio of the basin (see Table 1 in Antenucci and Imberger[1]). From the dispersion relation in Eq. (1), the internal wave period T is obtained as:

$$T = \frac{2\pi/f}{a_0 + a_1 S + a_2 S^2 + a_3 S^3}. \tag{2}$$

The phase speed of a non-rotating seiche of vertical mode *n*, c_n, for a given stratification profile can be estimated by solving the Taylor-Goldstein equation:

$$\frac{\partial^2 \phi(z)}{\partial z^2} + \left(\frac{N^2(z)}{(\overline{U}(z) - c_n)^2} - \frac{\partial^2 \overline{U}/\partial z^2}{\overline{U}(z) - c_n} - k^2 \right) \phi = 0, \tag{3}$$

where $\phi(z)$ denotes the vertical structure or streamfunction, $\overline{U}$ the background horizontal current, c_n the phase speed of vertical mode *n*, and k the horizontal wavenumber. $N^2(z) = -g\rho_0{}^{-1}(\partial\rho/\partial z)$ is the squared buoyancy frequency and ρ_0 a reference density. At the lake bottom and surface, $\phi(z = 0) = \phi(z = -D) = 0$.

Equation (3) was solved for a typical mean summer temperature (stratification) profile, referred to as the realistic temperature profile, T_r (Figure 1j in the main text), using the MATLAB code provided by Smyth[2], with $\overline{U} = 0$ (no background current). The horizontal wavenumber, k_m, corresponding to horizontal mode m, was set to $k_m = m\,\pi/L$, where L is the basin length[3].

Water density as a function of temperature was computed with the 25-term equation-of-state of McDougall et al.[4] with salinity kept constant at 0.03 psu; salinity plays a minor role in determining water density in Lake Geneva.

From Eq. (3), the non-rotating V2 and V3 phase speeds for the realistic temperature profile, T_r, are $c_2 \approx 21$ cm s^{-1} and $c_3 \approx 13.5$ cm s^{-1}, respectively. At 100-m depth, the main basin has a length of ~40 km and a width of ~10 km, which gives an aspect ratio of ~ 0.25.

Finally, with Burger numbers $S_2 = c_2/(Lf) \approx 0.1$ and $S_3 \approx 0.06$, the V2 and V3 Poincaré wave periods (Eq. 2) for the first horizontal mode are $T_{P,V2} \approx 14.7$ h and $T_{P,V3} \approx 15.7$ h, respectively (see Table 1 in Antenucci and Imberger[1] for an anticyclonic wave with aspect ratio 1/3 and $S \approx 0.10$ and $S \approx 0.06$). The estimated V2 and V3 Poincaré wave periods are in good agreement with the periods around 13.5-15.5 h observed and modeled during the summers of 2021 and 2022, respectively (Figures 1b, c and 4a). These results suggest that previously undetected V2 and V3 Poincaré waves caused the dominant current signal in the near-inertial band in the lake's deepest layers.

Supplementary Table 1. Details of the mooring deployed at ~305-m depth in the center of Lake Geneva (for location, see Figure 1a). For the present study, measurements from 1 June to 1 September in 2021 and 2022 are used.

	**Instrument depth [m]**	**Instruments and settings**
**2021 Field campaign**	255-300	10 RBRsolo T temperature loggers, vertical spacing 5 m, sampling interval 20 sec
	280	Downward-looking Nortek Signature 1000 (1000 kHz) Acoustic Doppler Current Profiler (ADCP): 29 bins of 1 m, 7 min ensemble interval, 1200 pings per ensemble.<br>Note: Due to low backscatter in the deepest layers, the last ~5-10 m above the lakebed often showed poor signal quality and were not used.
**2022 Field campaign**	53	Upward-looking Teledyne RDI Sentinel V (500 kHz) ADCP: 32 bins of 2 m, 20 min ensemble interval, 300 pings per ensemble.
	294	Upward-looking Teledyne RDI Workhorse Quartermaster (150 kHz) ADCP: 38 bins of 8 m, ~8 min ensemble interval, 130 pings per ensemble.

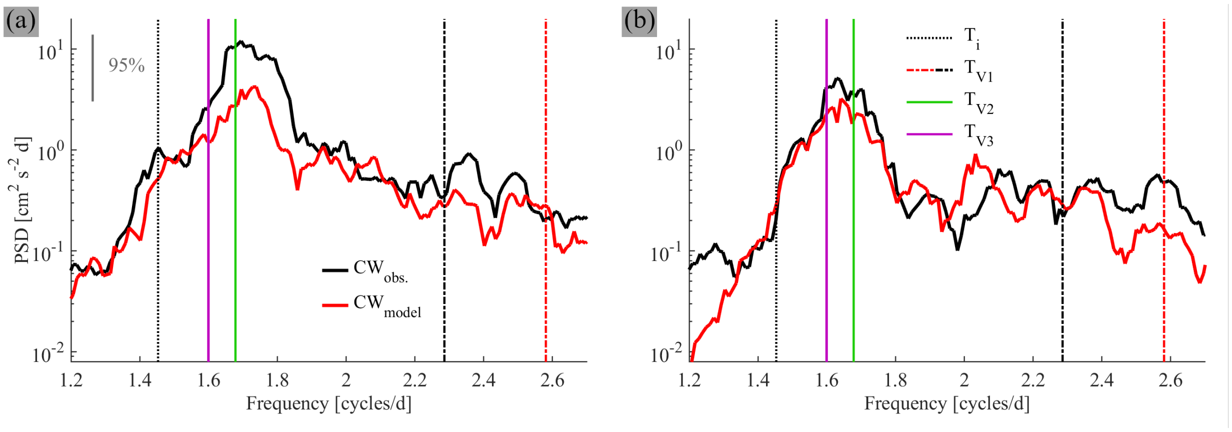

Supplementary Figure 1. Close-ups of the near-inertial band of the measured (black solid) and modeled (realistic simulations; red solid) clockwise rotary current spectra at the mooring location (depth-averaged over the lowest ~20 m) from 1 June to 1 September (a) 2021 and (b) 2022. The full frequency range is shown in Figure 1b, c in the main text. Vertical lines (left to right): Black dotted line: Inertial period (T_i = 16.5 h); Magenta solid line: V3 Poincaré period (T_{V3} = 15.0 h); Green solid line: V2 Poincaré period (T_{V2} = 14.3 h); Black dash-dotted line: V1 Poincaré period (T_{V1} = 10.5 h, as reported in Lemmin et al.[5]) and Red dash-dotted line: V1 Poincaré period (T_{V1} = 9.3 h, as determined in this study based on the idealized modeling results for summer 2022; see also Figure 4a). The 95% confidence interval is given in (a). Note that the V1, V2 and V3 Poincaré wave periods reported here were determined based on the idealized modeling results for summer 2022 as described in section, *V1, V2 and V3 Poincaré wave features revealed by idealized simulations* in the main text. These wave periods change depending on the stratification and, thus, can vary between different (summer) months and years. The latter explains the "mismatch" between the location of the near-inertial peaks in the current spectra for summer 2021 (panel a) and the V2 and V3 wave periods determined for summer 2022 (compare near-inertial peaks and green and magenta lines in panels a and b; see also Figure 1c, b).

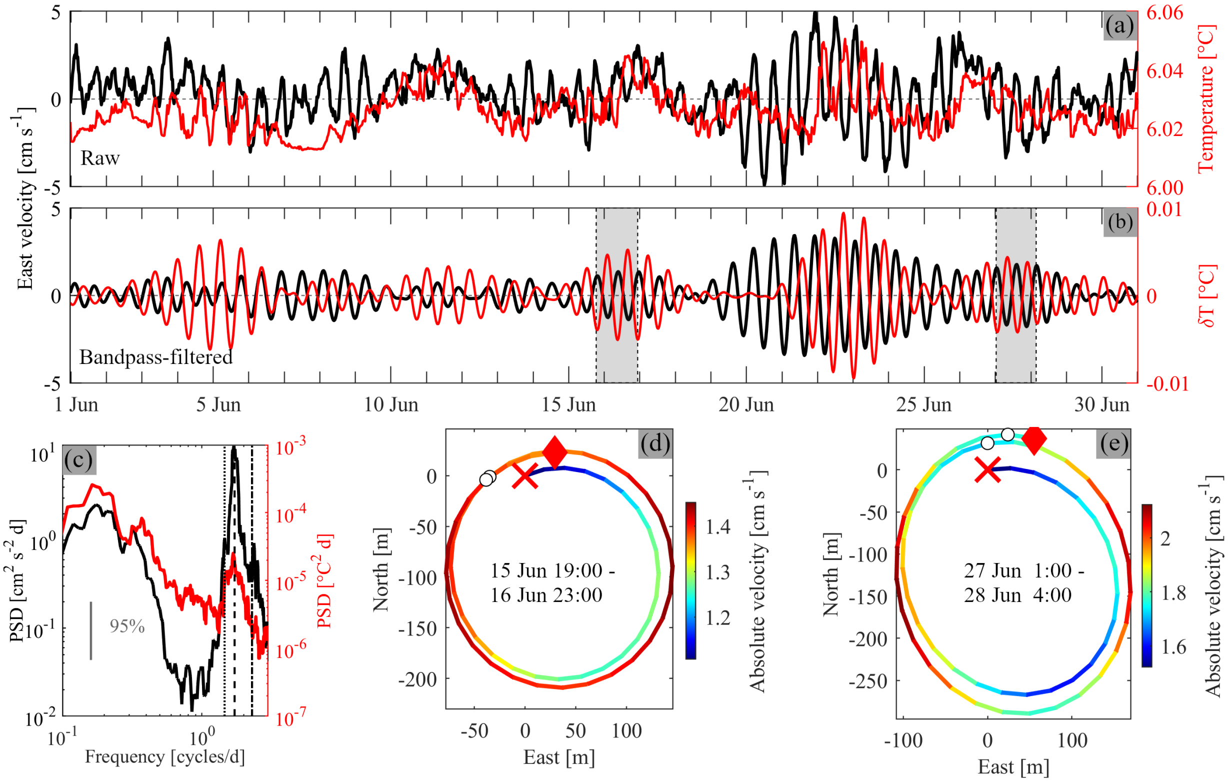

Supplementary Figure 2. Analysis of field data: (a) and (b) Raw and bandpass-filtered (13-15 h) east velocities depth-averaged over the lowest ~20 m (black) and temperatures (temperature variations, δT, in panel b) 15 m above the lakebed (red) (same as Figure 3b, c). (c) Clockwise rotary current spectrum depth-averaged over the lowest ~20 m (black; same as in Figure 1b) and temperature spectrum 15 m above the lakebed (red) from 1 June to 1 September 2021. Black dashed line: 14 h. Black dotted line: Inertial period 16.5 h. Black dash-dotted line: Vertical mode-one (V1) Poincaré period 10.5 h[5]. The 95% confidence interval is given. (d) and (e) Progressive vector diagrams of the bandpass-filtered (13-15 h), depth-averaged (lowest ~20 m) velocities around 15 and 27 June, respectively (see gray shaded areas in panel b). Red cross: start. Red diamond: end. Black circles are given every 14 h. All panels show measurements taken at the mooring location in 2021 (for location, see Figure 1a).

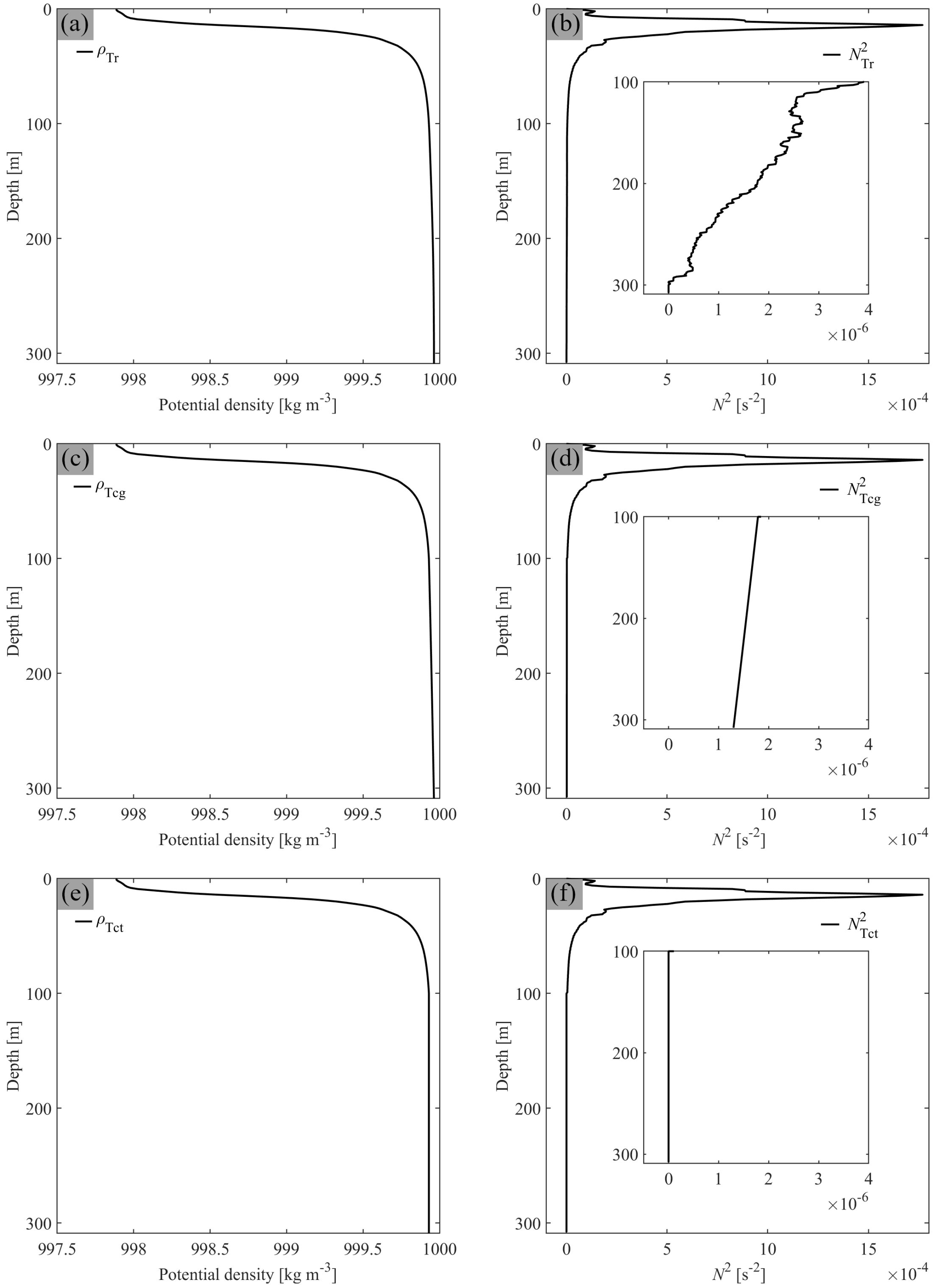

Supplementary Figure 3. Left column (a), (c), (e): potential density referenced to the atmospheric pressure computed with the equation-of-state of McDougall et al.[4] Right column (b), (d), (f): squared buoyancy frequency for different initial temperature profiles (see legends; cf. Methods section in main

text). The inset plots show close-ups below 100-m depth. T_r: temperature profile from the realistic simulations. T_{cg}: same as T_r, but with constant hypolimnetic temperature gradient below 100-m depth. T_{ct} same as T_r, but with constant hypolimnetic temperatures below 100-m depth.

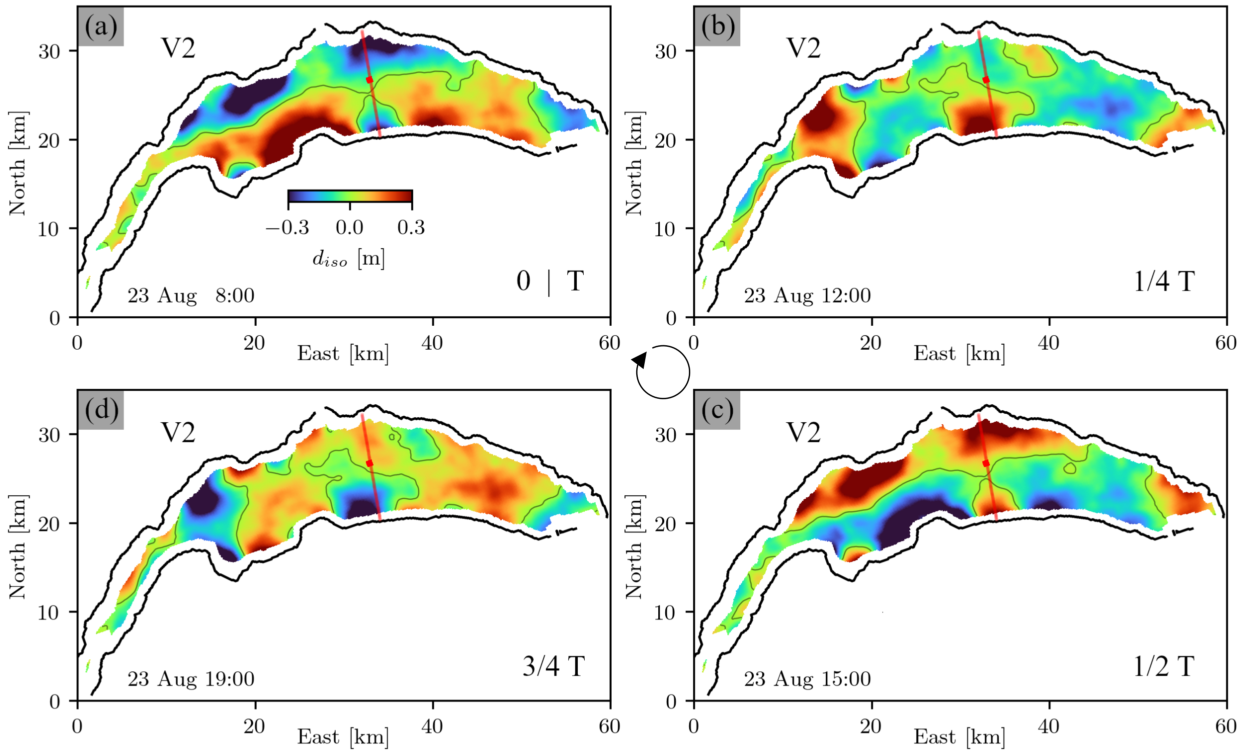

Supplementary Figure 4. Horizontal structure of the vertical mode-two (V2) Poincaré wave for the case of realistic stratification (idealized simulation for 2022) in the deep hypolimnion, profile T_r. (a) to (d): Quarter-cycle stages of vertical isotherm displacements (d_{iso}) at ~35-m depth over one V2 Poincaré wave period (T). Black lines: zero d_{iso} isolines. Red line: location of transect through the mooring location, see Figure 5a-d in the main text. The model was forced with *Bise* wind (Supplementary Figure 10a, b, e) and initialized with temperature profile T_r (Figure 1j in the main text). Results are bandpass-filtered (14.05-14.55 h) and a horizontal moving average over a window of ~1 km was applied. Red dot: mooring location.

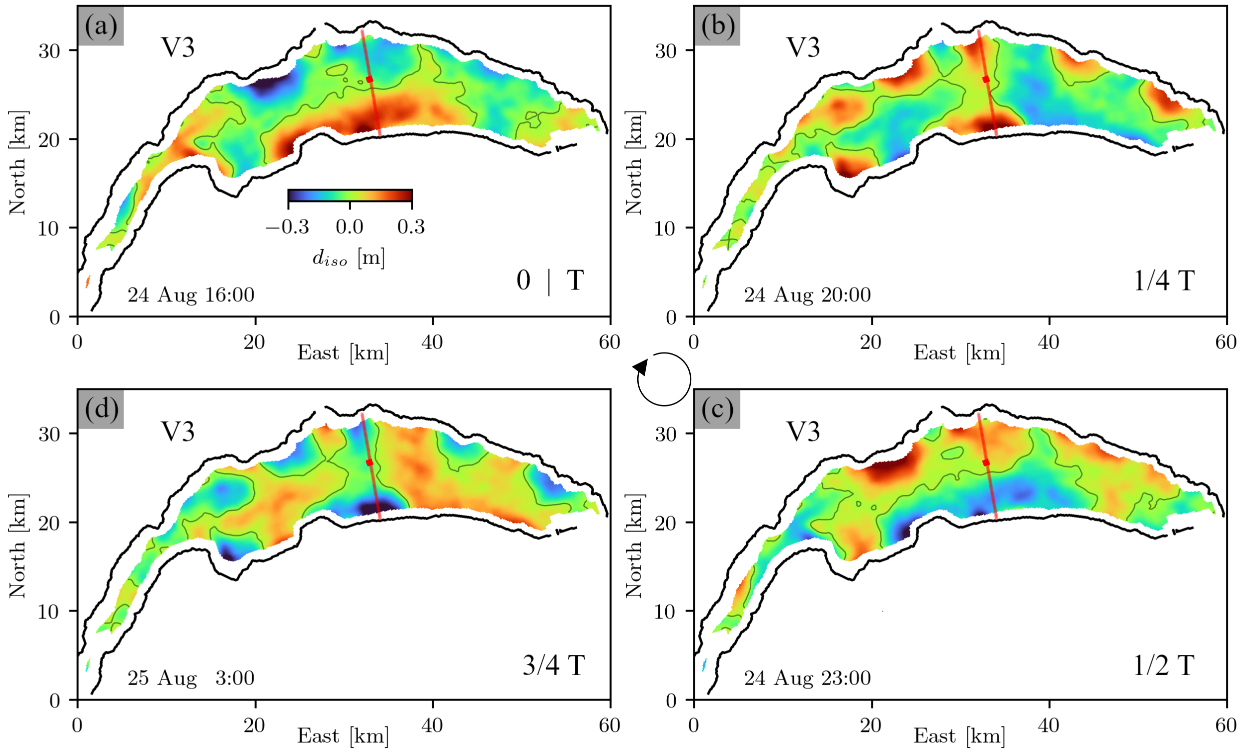

Supplementary Figure 5. Horizontal structure of the vertical mode-three (V3) Poincaré wave for the case of realistic stratification (idealized simulation for 2022) in the deep hypolimnion, profile T_r. (a) to (d): Quarter-cycle stages of vertical isotherm displacements (d_{iso}) at ~35-m depth over one V3 Poincaré wave period (T). Black lines: zero d_{iso} isolines. Red line: location of transect through the mooring location, see Figure 5a-d in the main text. The model was forced with *Bise* wind (Supplementary Figure 10a, b, e) and initialized with temperature profile T_r (Figure 1j in the main text). Results are bandpass-filtered (14.75-15.25 h) and a horizontal moving average over a window of ~1 km was applied. Red dot: mooring location.

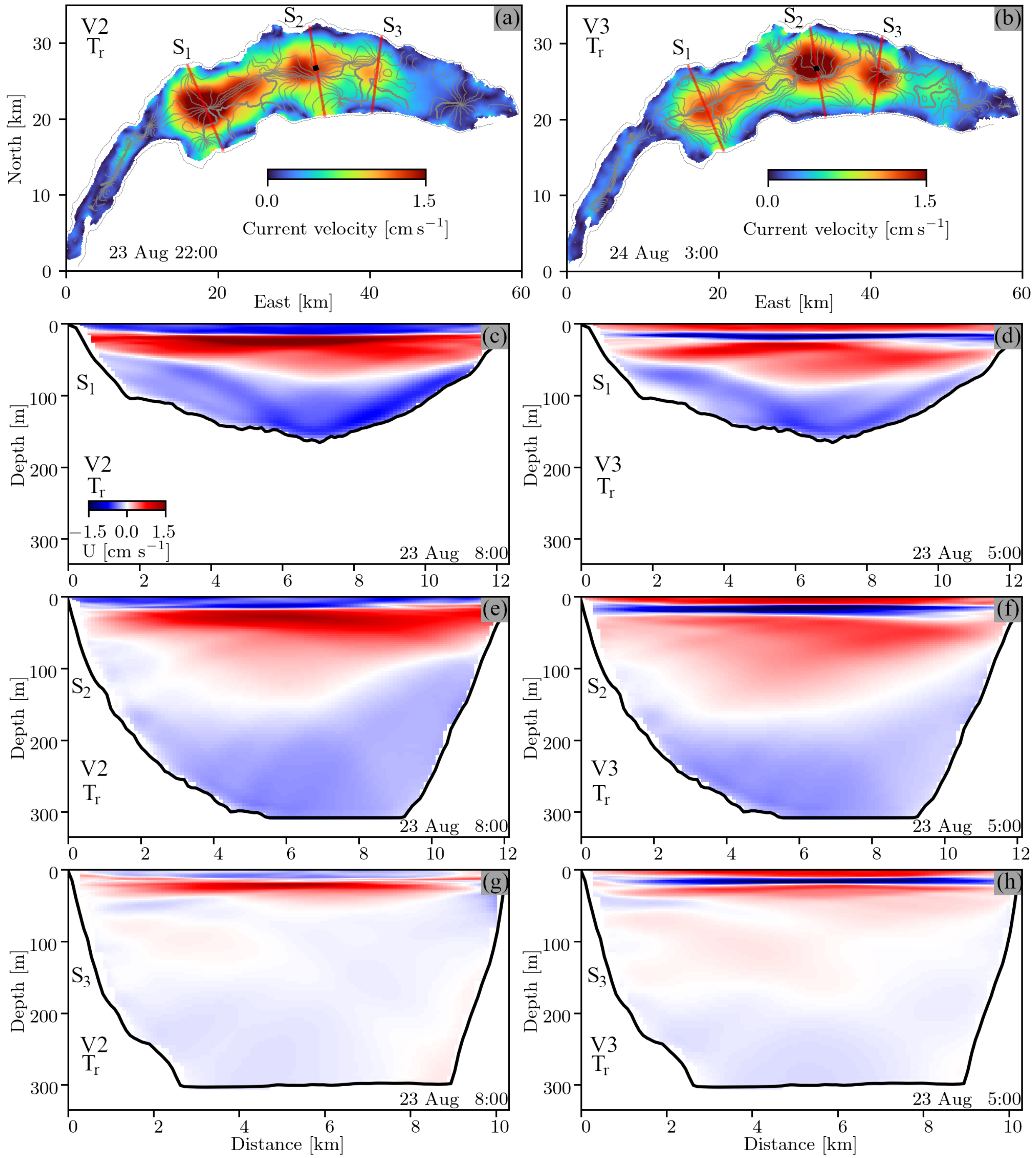

Supplementary Figure 6. Horizontal and vertical structure of the vertical mode-two (V2; left column) and vertical mode-three (V3; right column) Poincaré waves for the case of realistic stratification in the deep hypolimnion, profile T_r. (Idealized simulation for 2022). (a, b) Timelines of vertical isotherm displacement (d_{iso}) at ~35-m depth (grey lines) and depth-averaged (V2: 20-25 m, and V3: 15-20 m) current speeds (colors). d_{iso} timelines show the crest arrival time during one wave period (1-h intervals). Black dot: mooring location. (c-h) Current velocities along transects S1-S3 (red lines in (a, b)). Colors:

Currents into (red) and out of (blue) the plane. Distance is from the northern shore. The model was forced with *Bise* wind (Supplementary Figure 10a, b, e) and initialized with temperature profile T_{ct} (Figure 1j in the main text). Results are bandpass-filtered (V2: 14.05-14.55 h, and V3: 14.75-15.25 h). Transect S2 passes through the mooring location.

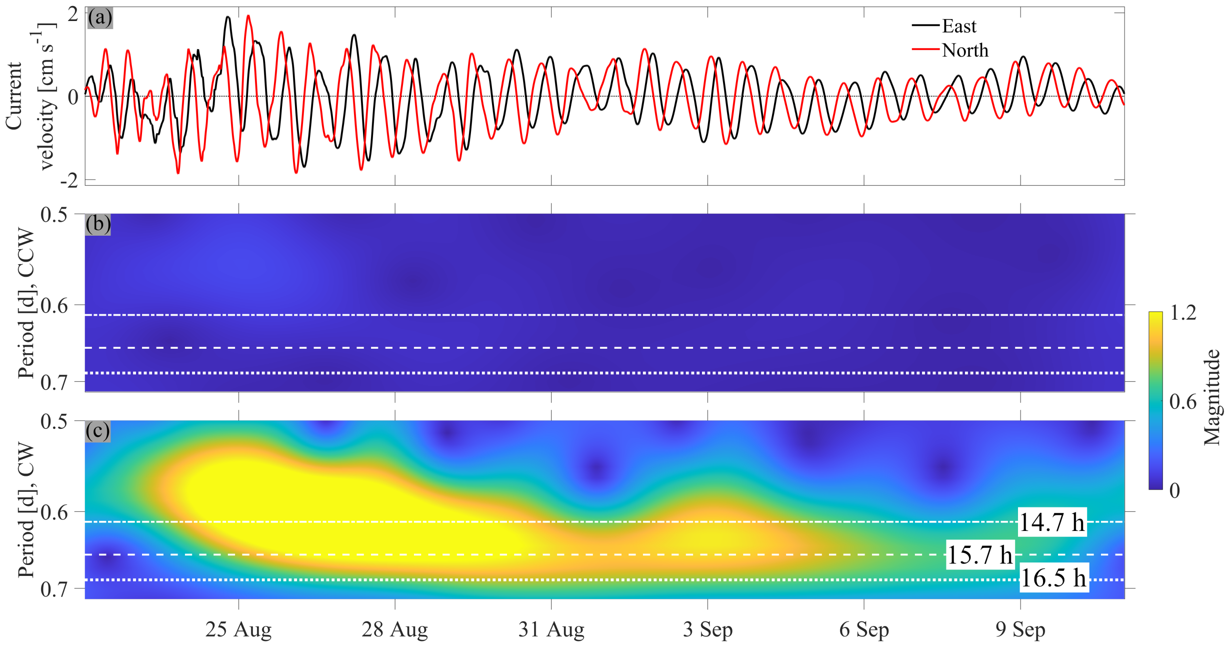

Supplementary Figure 7. (a) Modeled east (black) and north (red) velocities at the mooring location (white dot in Figure 1a) at 290-m depth (idealized simulation for 2022). (b) Corresponding counterclockwise (CCW), and (c) clockwise (CW) rotary wavelet transforms. The horizontal white lines mark periods of 14.7 h (theoretical V2 Poincaré wave period, see Supplementary Text 1; dash-dotted), 15.7 h (theoretical V3 Poincaré wave period, see Supplementary Text 1; dashed), and 16.5 h (inertial period; dotted). The model was forced with a 4-h long *Bise* wind impulse (Supplementary Figure 10a, b, e) and initialized with temperature profile T_r (Figure 1j in the main text). Note that with time after the wind impulse, energy is shifted from the theoretical V2 to the V3 Poincaré wave period, and it approaches the inertial period.

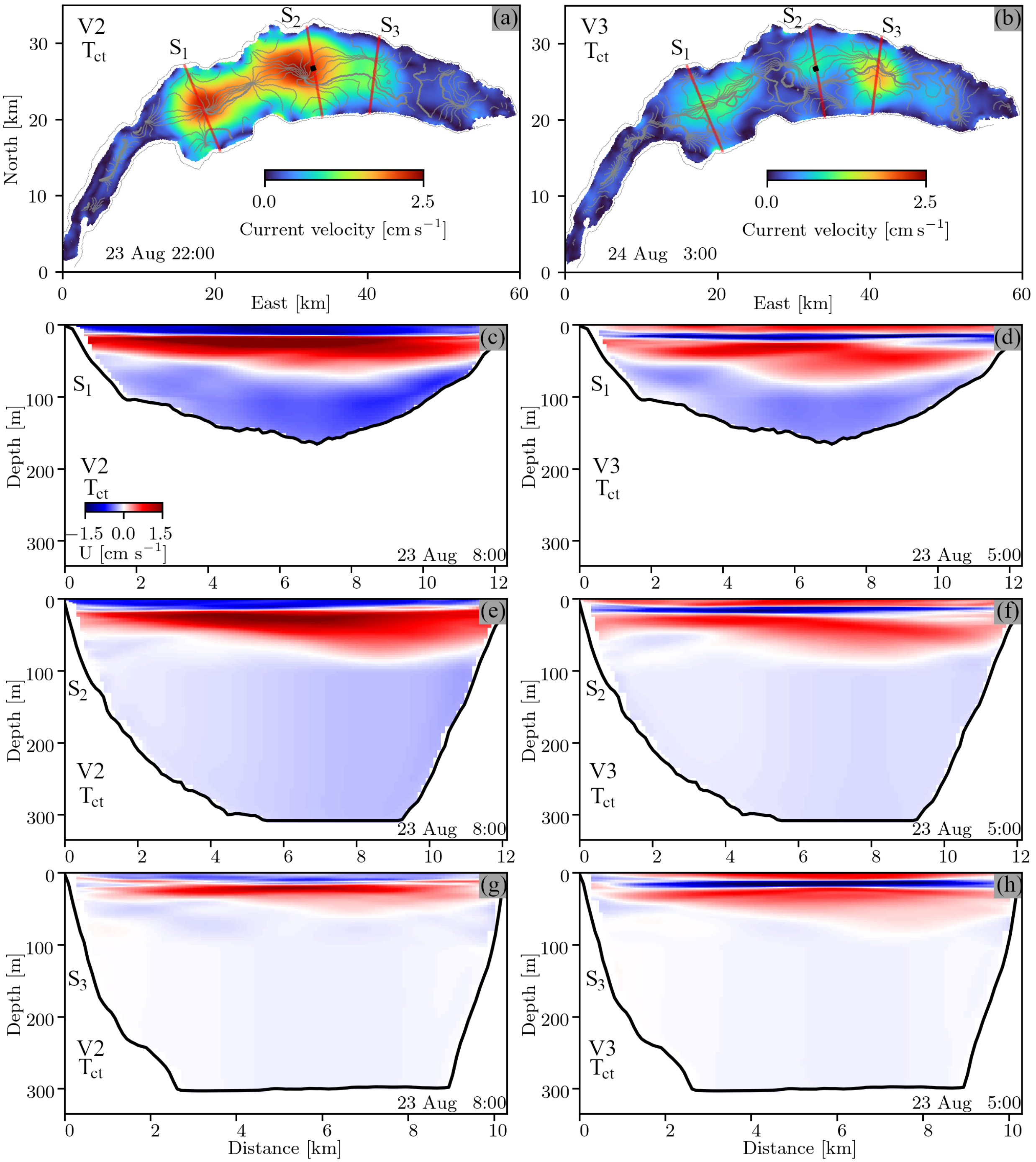

Supplementary Figure 8. Horizontal and vertical structure of the vertical mode-two (V2; left column) and vertical mode-three (V3; right column) Poincaré waves for the case of no stratification in the deep hypolimnion, profile T_{ct} (Idealized simulation for 2022). (a, b) Timelines of vertical isotherm displacement (d_{iso}) at ~35-m depth (grey lines) and depth-averaged (V2: 20-25 m, and V3: 15-20 m) current speeds (colors). d_{iso} timelines show the crest arrival time during one wave period (1-h intervals). Black dot: mooring location. (c-h) Current velocities along transects S1-S3 (red lines in (a, b)). Colors:

Currents into (red) and out of (blue) the plane. Distance is from the northern shore. The model was forced with *Bise* wind (Supplementary Figure 10a, b, e) and initialized with temperature profile T_{ct} (Figure 1j in the main text). Results are bandpass-filtered (V2: 14.05-14.55 h, and V3: 14.75-15.25 h). Transect S2 passes through the mooring location. Compare with Supplementary Figure 6.

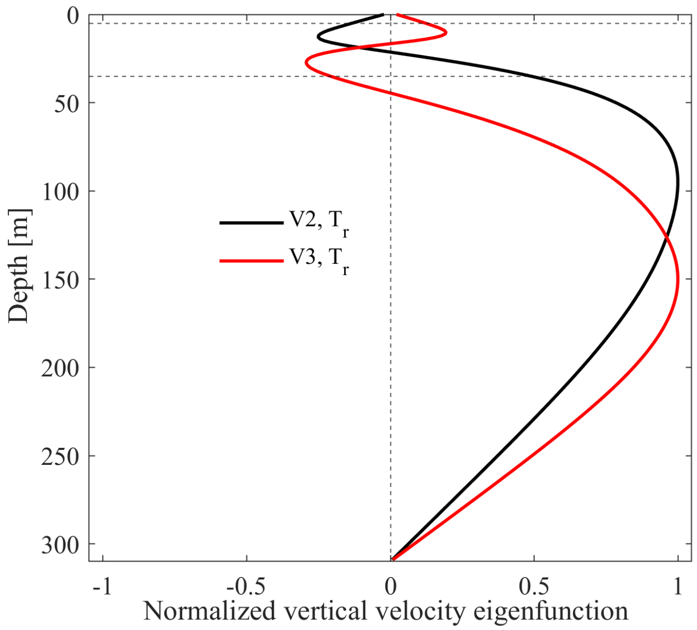

Supplementary Figure 9. Theoretical vertical velocity eigenfunctions of the vertical mode V2 (solid black line) and V3 (solid red line) Poincaré wave for the realistic temperature profile T_r (Figure 1j in the main text), normalized by the maximum value. The eigenfunctions were obtained by solving the Taylor-Goldstein equation without background current (for details, see Methods section). The thermocline is located between the two top horizontal dashed lines. Compare with Figure 5e, f.

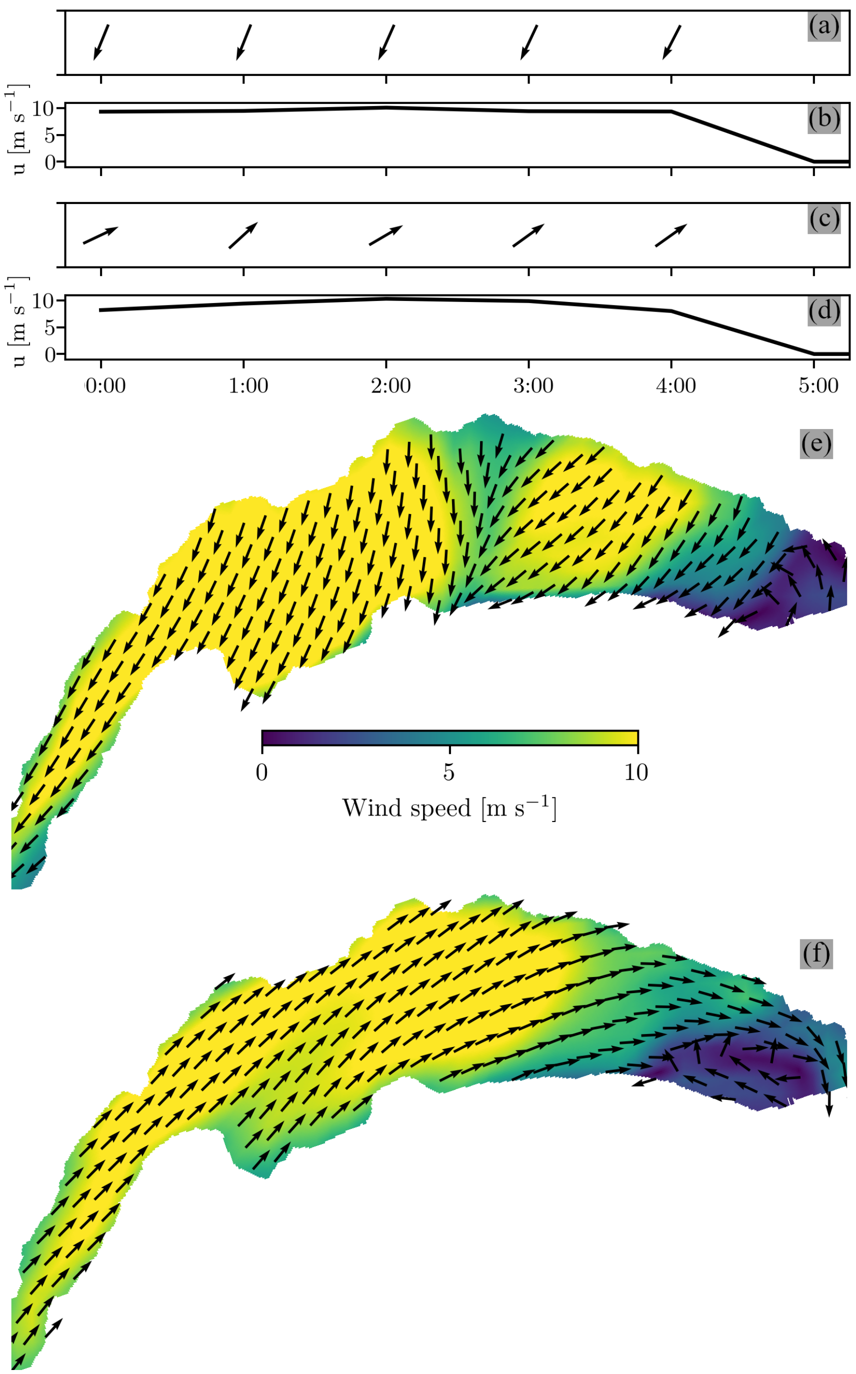

Supplementary Figure 10. (a) Wind direction and (b) wind speed from the MeteoSwiss COSMO-1 model (averaged over the main basin of Lake Geneva) during the 4-h long *Bise* wind impulse used to force the idealized simulations (for details, see Methods section in main text). (c) and (d) same as (a, b) but for the *Vent* wind impulse. (e) and (f) Two-dimensional COSMO-1 wind field (10 m above the lake surface) of the *Bise* and *Vent* wind, respectively, time-averaged over the entire duration of the wind impulse. The normalized black arrows show the wind direction. Colorbar: wind speed. For clarity, not all wind vectors are shown. For *Bise* and *Vent* direction, see Figure 1a.

Supplementary Movie 1

This movie shows the results of idealized 3D numerical simulations for Lake Geneva from 22 August 2022 at 1:00 to 28 August 2022 at 00:00 (CET). Bandpass-filtered current velocities for vertical mode-two (V2) Poincaré waves along different transects are presented in the top row and vertical mode-three (V3) in the bottom row (for transect location, see Supplementary Figure 6a, b). Colors: Currents in- (red) and out of (blue) the plane. Black arrows: Transversal currents (colorbar and arrow scale in top-left panel). Distance is from the northern shore. The model was forced with *Bise* wind (Supplementary Figure 10a, b, e) and initialized with temperature profile T_r (Figure 1j in the main text). Compare with Figure 5 and Supplementary Figure 6.

The three-layer (V2) and four-layer (V3) current structures typical of V2 and V3 seiches are clearly visible in all transects. The depths of the lower nodal lines (white color separating regions of red and blue) and thus the thickness of the current layers in the hypolimnion that flow in different directions vary significantly along the transects, with a maximum nodal depth of ~150 m in the deepest regions of the lake (transect S2; for details, see section, *Horizontal and vertical structure of V2 and V3 Poincaré waves* in the main text). Note also that the bottom currents grow stronger during the first few days, especially at transects S1 and S2, as can be seen by comparing the length of the arrows near the bottom over time (for details, see section, *Relative strength of V1, V2, and V3 Poincaré waves* in the main text).

Supplementary References

1. Antenucci, J. P. & Imberger, J. Energetics of long internal gravity waves in large lakes. *Limnology and Oceanography* **46**, 1760–1773 (2001).
2. Smyth, W. D., Moum, J. N. & Nash, J. D. Narrowband oscillations in the Upper Equatorial Ocean. Part II: Properties of shear instabilities. *Journal of Physical Oceanography* **41**, 412–428 (2011).
3. Hutter, K., Wang, Y. & Chubarenko, I. P. *Physics of Lakes Volume 2*. vol. 2 (Springer Berlin Heidelberg, Berlin, Heidelberg, 2011).
4. McDougall, T. J., Jackett, D. R., Wright, D. G. & Feistel, R. Accurate and computationally efficient algorithms for potential temperature and density of seawater. *Journal of Atmospheric and Oceanic Technology* **20**, 730–741 (2003).
5. Lemmin, U., Mortimer, C. H. & Bäuerle, E. Internal seiche dynamics in Lake Geneva. *Limnology and Oceanography* **50**, 207–216 (2005).
