# Supplementary material for: Strong bottom currents in large, deep Lake Geneva generated by higher vertical-mode Poincaré waves
Source: Commun Earth Environ. 2024 Sep 3;5(1):480. doi: 10.1038/s43247-024-01653-8 (PMC11371635; doi:10.1038/s43247-024-01653-8)
Supplement: Supplementary file 3 — Description of Additional Supplementary Files [file 43247_2024_1653_MOESM3_ESM.pdf]

Description of Additional Supplementary Files

File name: Supplementary Movie 1

Description: This movie shows the results of idealized 3D numerical simulations for Lake Geneva from 22 August 2022 at 1:00 to 28 August 2022 at 00:00 (CET).
